# Tailored axillary surgery – A novel concept for clinically node positive breast cancer

**DOI:** 10.1016/j.breast.2023.03.005

**Published:** 2023-03-08

**Authors:** Martin Heidinger, Michael Knauer, Christoph Tausch, Walter P. Weber

**Affiliations:** a Breast Center, University Hospital Basel, Basel, Switzerland; b University of Basel, Basel, Switzerland; c Breast Center Eastern Switzerland, St. Gallen, Switzerland; d Breast Center Zurich, Zurich, Switzerland

**Keywords:** Breast cancer, Breast surgery, Axillary lymph node dissection, Sentinel lymph node procedure, Axillary staging, Tailored axillary surgery

## Abstract

Axillary surgery in patients with breast cancer has been a history of de-escalation; however, surgery for clinically node-positive breast cancer remained at the dogmatic level of axillary lymph node dissection (ALND). In these patients, currently the only way to avoid ALND is neoadjuvant systemic treatment (NST) with nodal pathologic complete response (pCR) as diagnosed by selective lymph node removal. However, pCR rates are highly dependent on tumor biology, with luminal tumors being most present yet showing the lowest pCR rates. Therefore, the TAXIS trial is investigating whether in clinically node-positive patients, either with residual disease after NST or in the upfront surgical setting, ALND can be safely omitted. All patients undergo tailored axillary surgery (TAS), which includes removal of the biopsied and clipped node, the sentinel lymph nodes as well as all palpably suspicious nodes, turning a clinically positive axilla into a clinically negative. Feasibility of TAS was recently confirmed in the first pre-specified TAXIS substudy. TAS is followed by axillary radiotherapy to treat any remaining nodal disease. Disease-free survival is the primary endpoint of this non-inferiority trial, and morbidity as well as quality of life are the main secondary endpoints, with ALND being known for having a relevant negative impact on both. Currently, 663 of 1500 patients were randomized; accrual completion is projected for 2025. The TAXIS trial stands out in including clinically node-positive patients in both the neoadjuvant and upfront surgery setting, thereby investigating surgical de-escalation at the far-end of the risk spectrum of patients with breast cancer.

## Highlights

• Axillary lymph node dissection is currently indicated in most patients with clinically node-positive disease.
• The TAXIS trial investigates the de-escalated concept of tailored axillary surgery.
• Tailored axillary surgery reduces the tumor load in the axilla to the point where radiation should be able to control it.
• TAXIS accrual completion is projected for 2025 and primary endpoint analysis for 2029.

## 1 Introduction

Breast cancer surgery has been a history of de-escalation, ever since Halsted's maximization of loco-regional treatment began to be questioned over the 20th century [1,2]. However, surgery of the lymphatic system remained at the dogmatic niveau of axillary lymph node dissection (ALND) under the premises of staging information, local control, and survival maximization until the 1990's, when the sentinel lymph node (SLN) procedure was introduced for clinically node-negative breast cancer. Step by step the dogma was further weakened, with only few routine indications for ALND remaining in clinical practice [3]. The TAXIS trial investigates a novel surgical concept called “tailored axillary surgery” (TAS). It evaluates whether TAS reduces the tumor load to the point where adjuvant axillary irradiation can control it, and if this combination is non-inferior to ALND in terms of disease-free survival (DFS). The underlying hypothesis is, that less surgery-related morbidity improves the quality of life in patients with clinically node-positive breast cancer.

## 2 Early trials on de-escalation of axillary surgery

The first investigations regarding the omission of ALND were undertaken by Fisher et al. in the North-American NSABP-04 trial [4], followed by Louis-Sylvestre at Institut Curie in Paris, France [5] as shown in Table 1. The NSABP-04 trial investigated patients with operable breast cancer. On the one hand, clinically node negative patients were randomized to receive either radical mastectomy, total mastectomy, or total mastectomy and regional irradiation. No difference in 10-year disease-free survival, distant metastasis-free survival and overall survival was found. Even though locoregional recurrences occurred more often in patients receiving total mastectomy alone, no significant differences in survival outcomes between the experimental and control arms were seen. Patients with clinically positive axillary LN metastases were randomized to radical mastectomy or total mastectomy and regional irradiation. Similarly, no difference in locoregional recurrence, disease-free survival, distant metastasis-free survival and overall survival was detected [4]. The trial by Louis-Sylvestre et al. included patients with breast cancers ≤3 cm and clinically negative axillary lymph nodes (LN). Patients underwent breast conserving surgery with adjuvant breast irradiation and were randomly assigned to receive either ALND or axillary radiotherapy (ART). After a median follow-up of 15-years no differences in overall survival, disease-free survival, and local recurrence rates were noted. Axillary recurrences were seen statistically more often in the ART group, however with numerically marginal differences (ALND 1%, ART 3%) [5]. However, axillary staging information was still deemed necessary, leading to the development of the SLN procedure.

**Table 1:** Landmark trials informing the de-escalation of axillary surgery in breast cancer patients.

Trial	n patients	Recruitment Time Period	Inclusion criteria	Investigation	% of patients receiving ALND and having ≥1 positive LN (or additional positive LN in comparison to SLNB)	Recurrence rate		Overall survival rate	
						Treatment arm	Control arm	Treatment arm	Control arm
NSABP-04 [4]	1665	1971–1974	Operable breast cancer	If cN0: a) total mastectomy b) total mastectomy + regional irradiation c) radical Mastectomy (control) If cN1: a) total mastectomy + regional irradiation b) radical mastectomy (control)	n.A.	In cN0 10-year locoregional recurrence: a) 11.8% b) 4.6% In cN1 10-year locoregional recurrence:: 13.6%	In cN0 10-year locoregional recurrence: 6.9% In cN1 10-year locoregional recurrence: 14.7%	In cN0 10-year: a) 54% b) 59% In cN1 10-year: 39%	In cN0 10-year: 58% In cN1 10-year: 38%
Louis-Sylvestre et al. [5]	658	1982–1987	cT1 and cT2≤3 cm, cN0	Control: ALND Experimental: ART	21%	15-year local recurrence: 16.3%	15-year local recurrence: 17.2%	15-year: 75.5%	15-year: 73.8%
NSABP-032 [7,8]	5611	1999–2004	cN0, any T	Control: SLNB + ALND Experimental: SLNB - if positive SLN - ALND - if negative SLN - no ALND	38.6%	8-year loco-regional recurrence: 3.1%	8-year loco-regional recurrence: 3.1%	5-year: 95.0% (95%CI 94.0–96.0) 8-year: 90.3% (95%CI 88.8–91.8)	5-year: 96.4% (95%CI 95.6–97.2) 8-year: 91.8% (95%CI 90.4–93.3)
Z0011 [9]	891	1999–2004	cT1 and cT2 cN0	Control: SLNB with up to two metastases + ALND Experimental: SLNB with up to two metastases - no ALND	27.3%	10-year locoregional recurrence: 5.3%	10-year locoregional recurrence: 6.2%	10-year 86.3% (95%CI 82.2–89.5)	10-year: 83.6% (95%CI 79.1–87.1)
AMAROS [10]	1425	2001–2010	cT1 and cT2 cN0	Control: SLNB with metastasis + ALND Experimental: SLNB with metastasis - ART	33%	10-year locoregional recurrence: 3.8%	10-year locoregional recurrence: 3.4%	10-year: 81.4% (95%CI 77.9–84.4)	10-year: 84.6% (95%CI 81.5–87.1)
OTOASOR [11]	474	2002–2009	cT1 and cT2≤3 cm, cN0	Control: SLNB with metastasis + ALND Experimental: SLNB with metastasis - RNI	38.5%	8-year axillary recurrence: 1.7%	8-year axillary recurrence: 2.0%	8-year: 84.8%	8-year: 77.9%
IBCSG 23–01 [12]	931	2001–2010	cT1 and cT2, cN0	Control: SLNB + ALND Experimental: SLNB - if macrometasis - ALND - if micrometastasis - no ALND	13% in ALND group 3% in SLNB group with macrometastasis	5-year locoregional recurrence: 2.8% 10-year locoregional recurrence: 6.6%	5-year locoregional recurrence: 2.4% 10-year locoregional recurrence: 3.9%	5-year: 97.5% (95%CI 95.8–99.1) 10-year: 90.8% (95%CI 87.9–93.8)	5-year: 97.6% (95%CI 96.0–99.2) 10-year: 88.2% (95%CI 84.8–91.6)
AATRM [13]	233	2001–2008	cT1 and cT2≤3.5 cm, cN0	Control: SLNB with micrometastasis + ALND Experimental: SLNB with micrometastasis - no ALND	13.4%	5-year disease recurrence: 2.5%	5-year disease recurrence: 1%	n.A.	n.A.
SINODAR-ONE [14]	889	2015–2020	cT1 and cT2, cN0	Control: SLNB with up to two macrometastases + ALND Experimental: SLNB with up to two macrometastases – no ALND	44.0%	5-year recurrence free survival: 95.6% 5-year locoregional recurrence: 1.6%	5-year recurrence free survival: 96.4% 5-year locoregional recurrence: 0.9%	5-year: 98.8%	5-year: 98.9%

LN – lymph node, SLN – sentinel lymph node, SLNB – sentinel lymph node biopsy; CI – confidence interval; RNI – Regional nodal irradiation; ART – axillary radiotherapy; n.A. – not available.

## 3 Sentinel lymph node biopsy

In the 1990's, which is rather recently considering the long history of surgery for breast cancer, the feasibility of the SLN procedure was shown. This built the foundation for a much less invasive and morbid, yet oncologically safe surgical staging concept for the axilla. Veronesi et al. included 516 patients, of which 259 received SLNB and ALND only if the SLN was positive, which did not show a difference in overall survival after a median of 46 months. The pathological positivity rate was 33.9%, with an accuracy of 96.9%, as tested in the ALND group. In 34.3% (60/175) the positive SLN showed only micrometastases (i.e., <2 mm), whereas all of these patients received ALND, which subsequently found no more positive nodes in 83.4%, and only one more positive LN in 16.7% [6]. The technical outcomes in the NSABP B-32 trial showed a SLN removal rate of 97.2%. Interestingly, 3.9% of SLNs in the initial study were palpable only, showing a pathological positivity rate of 23.1%, compared with 9.9% in “hot” SLNs. Furthermore, in 61.4% of patients receiving ALND, the SLN was the only positive node identified [7]. When comparing patients receiving ALND only if SLN were positive with those receiving ALND irrespective of SLNB status, both locoregional recurrences as well as overall survival did not show significant differences [8] (Table 1).

Therefore, in clinically node-negative patients the SLN procedure was deemed applicable, and in case of negative SLN did not show an impact on recurrence-free or overall survival.

## 4 De-escalation in clinically node-negative, SLN-positive patients

In a next step, treatment de-escalation in patients with metastases in the SLN was investigated. Albeit methodological limitations have been criticized, the ACOSOG Z0011 trial remains a major landmark study in this regard. Patients with primary breast cancer ≤5 cm, palpably negative axillary LN, breast conserving surgery with adjuvant radiotherapy, and one or two SLN with metastases (i.e., micro- and macrometastases without gross extracapsular extension) in the removed lymph nodes were randomized to receive either ALND or no further axillary treatment. After 10 years, no differences in locoregional recurrence, disease-free and overall survival were noted [9] (Table 1). Similarly, the EORTC 10981–22023 AMAROS trial included patients with breast cancer up to 5 cm, clinically negative axilla, breast conserving surgery and whole breast irradiation or mastectomy, and tumor metastases in a SLN. Patients with either micro- (40% of patients) or macrometastases (60%) were randomly assigned to either ALND or axillary radiotherapy. After 10 years, no difference in axillary recurrence was noted, with strikingly low event rates in both groups, even though the comparison was formally underpowered. Furthermore no difference in overall survival, distant metastasis-free survival, and locoregional recurrence were reported [10]. (Table 1). Also, the OTOASOR trial reported on patients with breast cancer ≤3 cm, cN0, who received breast conserving surgery or mastectomy and showed metastasis in at least one SLN (60% macrometastasis, 34% micrometastasis, 6% isolated tumor cells). This cohort was randomized to receive either regional nodal irradiation (RNI) or ALND (whereas 23% received ALND followed by RNI). After 8 years, no difference in regional recurrence, disease free survival and overall survival were seen [11] (Table 1). The IBCSG 23–01 trial investigated, whether in patients with primary breast cancer ≤5 cm, breast conserving surgery or mastectomy, and one or more micrometastases (i.e., ≤2 mm without extracapsular extension) in the removed lymph nodes, could safely be spared ALND without any further therapy. After a median follow-up of 9.7 years, no differences in DFS was seen [12] (Table 1). The smaller AATRM trial confirmed these findings, randomizing patients with micrometastases in the SLN and breast conserving surgery or mastectomy for primary breast cancer ≤3.5 cm and clinically unremarkable nodal status to either ALND or no further axillary treatment. After a median follow-up of 5 years, no differences in disease-free survival was noted [13] (Table 1). Recently, the SINODAR-ONE trial has published 3-year follow-up data as the first ACOSOG Z0011 validation trial. Here, SLNB was shown to be non-inferior compared to ALND for both survival and relapse rates in patients with primary breast cancers up to 5 cm, and up to two macrometastatic LN [14] (Table 1). Importantly, the rate of axillary tumor-burden left behind, when omitting ALND was shown to be at least one positive lymph node in 27–44% as assessed by ALND in the control arms of the above-mentioned trials. Furthermore, ≥pN2 stage was present in 9.8–22% of patients undergoing ALND, with oncologic outcomes still not comprised [[9], [10], [11],^14^]. The number of retrieved SLN correlates with a decreased false-negative rate, which is overall around 17%, but can be decreased to <10% when three or more lymph nodes are removed. Importantly, 96.3% of lymph node metastases are identified when removing three SLN, compared to 99.1% once 5 SLN are removed [7,[15], [16], [17]]. However, even an accepted 5% false-negative rate did not worsen oncologic outcomes [16]. Furthermore, the number of removed SLN also correlates with a subjectively perceived increase of lymphedema, possibly due to sensory nerve injury [18].

These trials provided evidence that the combination of modern radiotherapeutic and systemic treatment approaches may sufficiently control and treat axillary disease, obviating the need for complete surgical removal.

The question, whether axillary surgery is at all necessary in selected patients with early breast cancer is addressed in the randomized-controlled SOUND trial, investigating the distant disease-free survival in patients with clinically node-negative primary BC < 2 cm, and the INSEMA trial, investigating invasive disease-free survival in clinically node-negative patients with primary BC < 5 cm. In both trials patients planning to undergo BCS and adjuvant radiotherapy are enrolled. Here, the experimental arm will forego any axillary intervention, with the control arm undergoing SLNB [19,20]. These, trials are therefore inspired by earlier ALND omission trials and specifically address the question if the SLN procedure can be replaced by ultrasound irrespective of age and subtype.

## 5 Axillary surgery after neoadjuvant treatment and pathologic complete nodal response

Advances in systemic therapy approaches for breast cancer have led to multiple neoadjuvant regimens, especially for Her2-positive and triple-negative breast cancer, showing pathologic complete response (pCR) rates of 58–67% [[21], [22], [23]], and therefore questioning the most appropriate extent of breast cancer surgery. Focusing on the axillary surgery of initially node-positive (cN+) patients, data from an exploratory analysis within the GeparOcto trial showed a breast pCR rate of 45.0%, of which 91.7% also showed axillary pCR [24]. Data confirmed by a Korean trial as well as a Canadian series, showing axillary pCR in breast pCR patients in 86.6% and 83.0% respectively [25,26]. Independent axillary pCR rates in initially cN + patients were investigated in a systematic review and meta-analysis including 57 531 patients, which reported rates of 13% for luminal A cancer, 18% for HR-positive/Her2 negative, 35% for luminal B, 45% for HR-positive/HER2 positive, 48% for triple negative, and 60% for HR-negative/Her2 positive cancers [27]. Recently, published data from the MARI trial reported axillary pCR rates of 9%, 59%, 94%, and 54% in luminal breast cancer, HR-positive/Her2-positive tumors, HR-negative/Her2-positive breast cancer, and triple-negative tumors, respectively.

Considering the omission of ALND in patients undergoing neoadjuvant systemic treatment (NST), the ACOSOG Z1071, the SENTINA trial, as well as the SN FNAC study provided information on the false-negative rate of the SLNB, which was 12.6%, 14.2%, and 8.4% (13.3% if considering isolated tumor cells as node-negative), respectively [[28], [29], [30]]. In light of these results, Caudle et al. reported a relevantly lower FNR of 2.0% in a pilot study of the same patient collective, using targeted axillary dissection (TAD), a novel technique combining SLNB with the selective removal of pre-NST clipped and histologically confirmed positive LN [31]. These results were validated in multiple studies, confirming feasibility and reproducibility of this method, with FNR of 4.3–9%, using multiple methods of clipped-node localization (iodine-seed, ultrasound guidance, tattoo) [[32], [33], [34], [35], [36], [37]].

Another technique is the MARI (marking axillary lymph nodes with radioactive iodine seeds) procedure, marking the largest tumor-positive LN (MARI node) pre-NST, which is subsequently selectively removed after NST. Furthermore, patients are staged with a FDG PET/CT before NST and classified to either <4 (cALN <4) or ≥4 (cALN ≥4) FDG-avid axillary lymph-nodes. After NST, patients are classified into ypMARI negative, or ypMARI positive. According to the study protocol, patients with less than 4 FDG-avid LN in pre-NST staging, and a negative MARI LN receive no further treatment. Those patients with either cALN ≥4 + ypMARI negative, or cALN <4 + ypMARI positive receive axillary radiotherapy, and patients with cALN ≥4 + ypMARI positive receive ALND. This method showed a FNR of 7% with a median of one removed LN, and a potential ALND-avoidance in up to 82% of patients, resulting in potential undertreatment in 3% [[38], [39], [40]]. After a median follow-up of 3-years, recurrence occurred in 5.4% (3/56 patients) with negative MARI-LN and cALN<4, and 9.3% (4/43) with MARI-negative, cALN≥4 receiving adjuvant ART. Based on this methodology, the RISAS (Radioactive Iodine Seed localization in the Axilla with Sentinel node procedure) trial was initiated, in which a positive lymph node is marked with an iodine seed before NST, and removed together with SLN after NST completion. Patients enrolled subsequently underwent completion ALND. Here, the FNR was reported at 3.5% [41].

Ongoing studies investigating oncological outcomes in cN + patients converting to ypN0 includes the OPBC-04 OMA study examining recurrence-rates in patients undergoing SLNB vs. TAD. The NSABP B-51 trial includes patients with T1-T3 breast cancer, presenting with histologically confirmed cN1, who undergo NST, breast conserving surgery or mastectomy, and are ypN0 after ALND, SLNB + ALND, or SLNB. These patients are randomized to receive either whole-breast irradiation (WBI), WBI and RNI after BCS, chest wall irradiation and RNI after mastectomy or no adjuvant radiotherapy (NCT01872975) [42,43]. Furthermore, the UK based ATNEC trial investigates, whether in patients with cT1-3 breast cancer and confirmed nodal-disease pre-NST, who convert to ypN0 confirmed by SLNB after NST, omission of axillary treatment is non-inferior to the control arm receiving either ALND or ART [44]. However, how to proceed in patients without nodal pCR or in the upfront surgical setting as e.g., in luminal breast cancers representing the majority of breast cancer with low pCR rates when using NST remains an open matter.

## 6 Axillary surgery in clinically node-positive patients

The only way to spare clinically node-positive patients ALND today is neoadjuvant systemic treatment and confirmed nodal pCR after selective LN removal as a diagnostic surgical procedure. The exception are patients with imaging-positive nodal disease that is non palpable, as almost half of these patients can undergo the SLN procedure without ALND according to the Z0011 protocol [45]. In light of the above-described advances, the question remains, whether in all clinically node-positive BC, both in the upfront surgery setting and in case of residual nodal disease after NST, a de-escalation of surgical therapy combined with an escalation of adjuvant radiotherapy may be warranted.

Retrospectively, real-life data from the national cancer database showed controversial results. In a series from 2006 to 2014, with patients showing cT1-3, cN1, cM0 breast cancer, who underwent NST and had residual disease in at least one of ≤4 removed LN (defined as SLNB) two matched cohorts were formed, one having received adjuvant RNI (n = 304), and a second having received ALND and RNI (n = 1313). Estimated 5-year OS results showed a significant benefit for patients with ALND (77%) over SLNB (71%), with a HR of 1.7 (95%CI 1.3–2.2). Interestingly, the analysis did not show an OS difference in patients with luminal A or B tumor, and only one affected LN (HR 1.03, 95%CI 0.59–1.8) [46]. A similar analysis covering 2012–2015 did not show a difference in OS, comparing matched cohorts receiving SLNB (n = 206) vs. ALND (n = 1205), with 5-year OS of 79% and 69% (p = 0.33) respectively [47].

Currently, three trials are addressing the question regarding de-escalation of axillary surgery in clinically node-positive patients. Firstly, the Alliance A011202 trial (NCT01901094) includes patients with cT1-3, cN1 breast cancer, undergoing NST followed by breast conserving surgery or mastectomy with SLNB. Patients with positive SLN are thereafter randomly assigned to undergo either ALND and extended nodal radiotherapy sparing the dissected axilla or extended nodal irradiation including the full axilla. So far, no results have been published.

The before-mentioned MARI trial, also includes patients with cALN <4 and positive ypMARI LN, undergoing adjuvant axillary radiotherapy, and patients with cALN ≥4 + ypMARI positive receiving ALND. The reported 3-year axillary recurrence-free interval was 98.2%. However, the 5 reported axillary recurrences all occurred in cALN <4 patients, whereas four occurred in ypMARI positive patients (3-year axillary recurrence rate 3.4%), which all had triple negative breast cancers [48], leading to calls for caution regarding the omission of ALND in patients with residual disease in this molecular subtype [49].

Furthermore, two registry based trials AXSANA (NCT04373655) [32] and MINIMAX (NCT04486495) [50] both include patients with or without axillary complete response after NST.

And finally, the TAXIS trial (NCT03513614), investigating patients with AJCC/UICC stage II-III and histologically confirmed node-positivity [51].

## 7 Tailored axillary surgery – a novel concept for clinically node positive breast cancer

To date, the only way to omit ALND in patients with clinically node-positive is to achieve nodal pCR after NST, as determined by limited axillary surgery, without removing all axillary LN. This strategy shows clear limitations, namely the necessity of NST in a cohort with mainly luminal tumor biology showing low pCR rates [27], and ALND as standard of care in residual disease. In order to find ways to avoid ALND in both the upfront surgery setting, as well as in patients with residual disease after NST, without compromising patient survival, TAXIS is built on the hypothesis, that residual, non-palpable nodal disease can be controlled by adjuvant radiotherapy with or without prior use of NST. Here, a novel surgical approach using a combination of several established surgical techniques is being tested, namely “tailored axillary surgery” (TAS), with the aim of selectively removing positive axillary LN to reduce the tumor load to the point where radiotherapy can control it. TAS therefore is not only a staging procedure to determine nodal pCR, but furthermore a therapeutic concept to selectively remove positive nodes in the adjuvant and neoadjuvant setting.

**Fig. 1:**
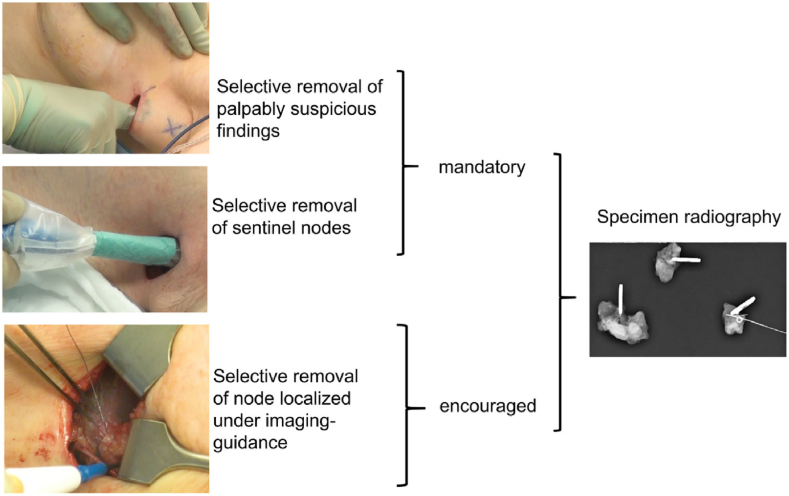
The concept of tailored axillary surgery (TAS) in the TAXIS trial [52].

Technically, the obvious axillary tumor burden is selectively reduced by combining the SLN procedure with the removal of palpably suspicious LN, thereby tailoring the extent of axillary surgery to the extent of axillary disease. Palpable disease is currently rarely encountered during the SLN procedure as it is one of its main contraindications. Another difference to the SLN procedure is the optional use of imaging-guided localization of clipped or suspicious nodes (Fig. 1). Clearly, the definition of palpably suspicious nodes is arbitrary, and hence, the concept of TAS is pragmatic and up to the discretion of the surgeon. The first prespecified subproject showed that TAS works inasmuch as it selectively reduced the number of positive nodes, while remaining much less radical than ALND.

**Fig. 2:**
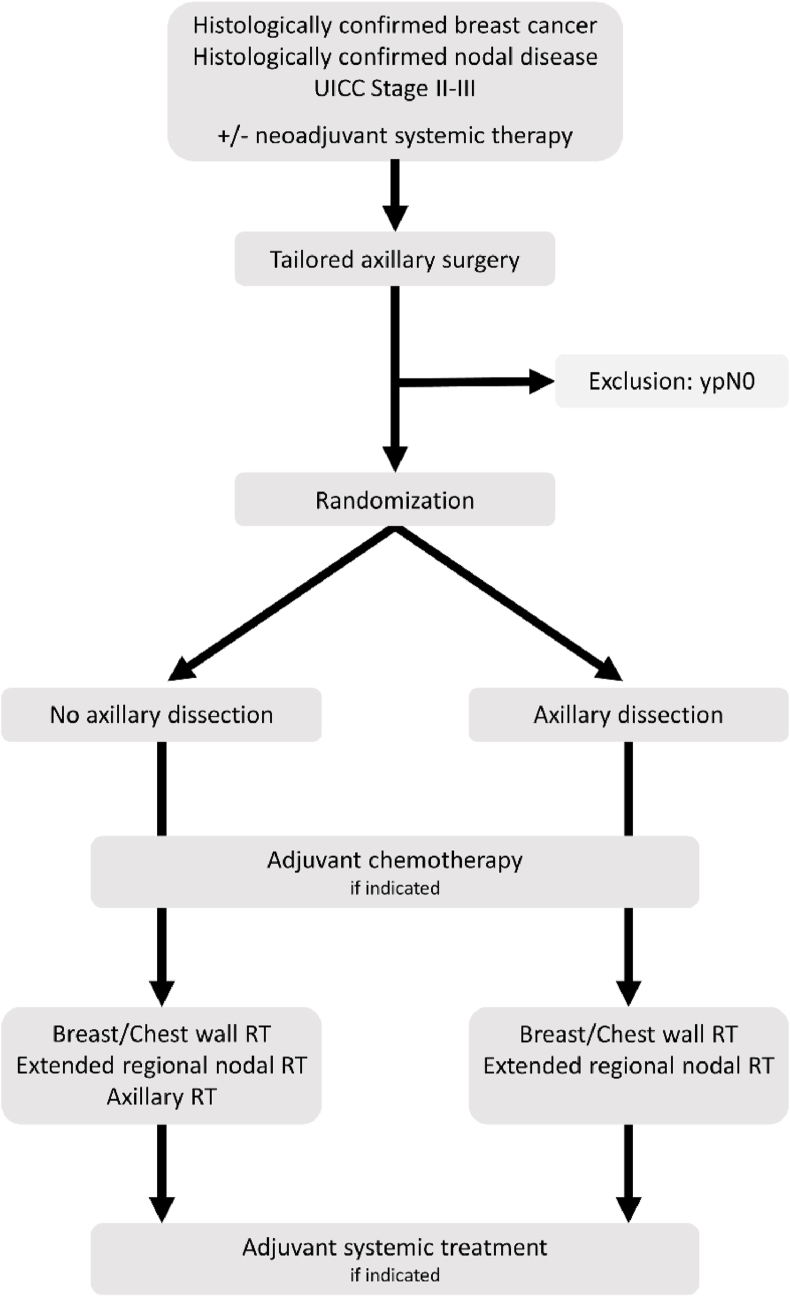
Study flowchart of the TAXIS trial, adapted according to Henke et al. [51]. ypN0 - nodal pathologic complete response after neoadjuvant systemic therapy; RT - radiotherapy.

For patients to be eligible after NST, residual disease has to be re-confirmed in the LN. In the upfront surgery setting, node-positivity has to be confirmed at diagnosis. Patients are randomly assigned after TAS and intraoperative confirmation of removal of the clipped lymph-node to receive either no further axillary surgery and RNI including the axilla, or to completion ALND and RNI without the axilla in the context of breast/chest wall irradiation. The primary endpoint studied in this non-inferiority trial is disease-free survival, with a projected analysis of the primary endpoint in 2029 [51]. A detailed study flow-chart is depicted in Fig. 2.

The tested hypothesis is, whether adjuvant RNI including a clinically positive axilla that was turned -by using TAS- into a clinically (macroscopically, grossly) negative axilla is non-inferior to ALND in terms of oncological outcomes, with less associated morbidity and increased QoL [51].

**Fig. 3:**
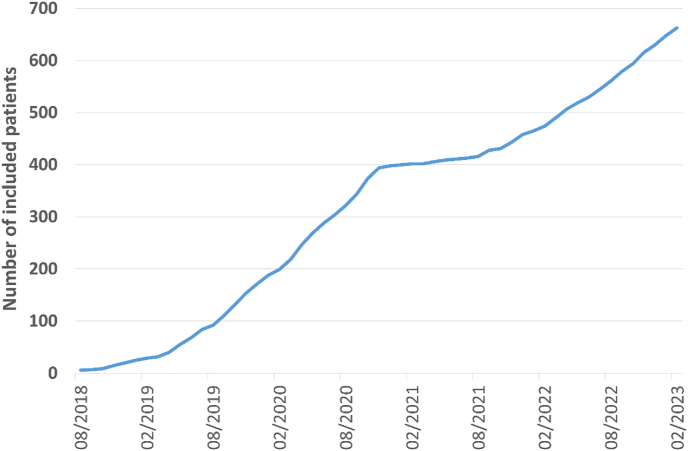
Accrual of the TAXIS trial.

Currently, 44 study centers in 6 countries are involved in the study implementation and patient recruitment. Accrual is again as planned, after a transfer of the study sponsor, showing 663 patients randomized by end of January 2023 (Fig. 3). The pragmatic concept of the TAXIS trial called for inclusion of the vast majority of patients with clinically node positive breast cancer who would have undergone ALND outside of the TAXIS trial as standard treatment. While a homogenous patient population of highly selected patients, for example by focusing on specific patient subgroups, tumor stages, subtypes or treatment settings (neoadjuvant versus adjuvant) would have allowed more precise risk estimates and sample size calculations, the results of the trial would have only been applicable to that specific patient population and generalizability would have been very limited, as it is in many explanatory clinical trials. Therefore, the risk estimates used for sample size calculation reflect the risk of the TAXIS patient population associated with clinically node-positive breast cancer. The study is powered for the primary endpoint analysis and not for preplanned subgroup analyses, including the neoadjuvant versus adjuvant setting. The total planned sample size to test non-inferiority of ART compared to ALND comprises 1500 patients based on statistical considerations of the primary endpoint DFS. The type I error was set at 5% with a power of 80%, requiring 385 events to show non-inferiority with a set non-inferiority margin of 1.289 (corresponding to a DFS at 5 years of 80% in the control arm, and 75% in the interventional arm) [[53], [54], [55]]. Patients are randomized in a 1:1 ratio, for a total of 750 patients per treatment group. Stratification factors include (i) responsible surgeon, (ii) type of positive node detection (imaging and non-palpable vs. palpable in upfront surgery setting vs. after NST), newly diagnosed vs. recurrence, normofractionated vs. hypofractionated radiotherapy, male vs. female. These stratification-factors are expected to further increase the study power.

The feasibility of TAS has recently been confirmed in a pre-planned substudy of 296 patients [52]. Of those, node-positivity was diagnosed in 51% by palpation, with the other half detected on imaging. Of 125 patients (42%) receiving NST, 71 (56.8%) showed nodal pCR. Tailored axillary surgery showed a clip removal rate of 94.3%. Clipping of the largest biopsy-proven LN was in 99% carried out using ultrasound, with the three most commonly used clip-types being Titanium/Stainless steel clips with gel (31.1%) or without gel (29.7%), and Nitinol ring markers (30.7%). The type of clip used was not associated with successful surgical removal of the clipped node in both, patients with residual nodal disease, and in nodal pCR. Image-guided clip localization was attempted in 257 patients (86.8%), being successful in 242 (94.2%), whereas 72% (185/257) of localizations were attempted preoperatively, and 28% (72/257) intraoperatively. Localization techniques preoperatively mainly included wire (50.3%), radioguided occult lesion localization (28.1%), and seed (14.1%), whilst intraoperatively wire localization (59.7%) was followed by ultrasound alone (29.2%), and tattoo (2.8%). Surgical removal of the clipped node was successful in 95% of patients with, and 92% without image-guided localization. A trend to less removed LN was observed in the group with image-guided localization (median 4, IQR 3–7) compared to those without (median 6, IQR 4–7; p = 0.09) [52]. To further investigate the precision of TAS with or without imaging-guided localization a substudy including the first 500 randomized patients is currently carried out.

In 225 patients with confirmed clinical node positivity, or residual nodal disease after NST, TAS was performed. In the upfront surgery setting, the median number of removed LN was 5 (IQR 3–7), of which a median of 2 LN (IQR 1–4) were positive. After NST a median of 4 LN (IQR 3–5) were removed, with a median of one positive LN (IQR 1–2). Subsequently, 100 patients underwent completion ALND, removing a median of 14 additional LN, whereas in 70% additional positive nodes were removed. The FNR of TAS in patients undergoing subsequent ALND was 1.8%, with a negative predictive value of 95.5% [52]. Long-term follow-up of the TAXIS trial will show whether treatment of the remaining nodal tumor load with RNI is oncologically non-inferior to ALND.

Interestingly, palpable nodal disease did not lead to higher postoperative pN stages compared to imaging findings in the upfront surgery setting (pN1 in 36.4% vs. 32.4% respectively) [52]. These findings are in line with two smaller series of cN1 patients with upfront surgery, showing a pN1 rate of 44.6% in a cohort of 91.5% ER-positive patients [56], as well as a second cohort of HR+/Her2 negative patients with palpable nodal-disease, undergoing ALND, showing 43% of cases with two or fewer affected LN [57]. These studies questioned the assumption that palpable disease indicates a higher tumor load than imaging-detected.

Accrual completion of the TAXIS trial is projected for 2025, with the primary endpoint analysis expected in 2029.

## 8 Is axillary dissection necessary for adjuvant treatment decisions in node-positive breast cancer?

De-escalation of axillary surgery, especially in node-positive patients challenges established criteria for adjuvant therapy (i.e., chemotherapy in patients with ≥4 positive LN in luminal BC), as well as recently investigated patient subgroups with LN-based cut-off values for the decision on both systemic as well as local therapies, and even response-driven therapeutic decisions after NST. A recent review addressed the impact of the monarchE and RxPONDER trials on axillary surgery [58]. Specifically, the monarchE trial randomly assigned high-risk (e.g., ≥4 positive LN, or 1–3 positive LN with either tumor size ≥5 cm, histologic grade 3, or Ki-67 ≥ 20%) HR+/Her2-patients to receive standard endocrine therapy with or without Abemaciclib, whereas the addition of this CDK4/6 inhibitor showed significantly improved 2-year invasive disease-free survival [59]. The question remains, whether patients with 1–3 positive SLN but no additional risk factors should undergo ALND, to determine, whether ≥4 positive LN are present. Mittendorf et al. concluded that systemic trials should be interpreted in light of recommended, evidence-based surgical therapies, whereas the omission of ALND in patients meeting monarchE inclusion criteria may partly lead to understaged but rarely undertreated patients, leaving the authors to recommend that routine ALND is not indicated to evaluate eligibility of the monarchE protocol [58]. Furthermore, the question whether completion ALND might have an influence on adjuvant treatment is also relevant when considering the inclusion criteria of the RxPONDER and Mindact trials investigating molecular tumor markers to refine chemotherapy indications [60,61]. In both trials patients with a nodal-burden of up to three positive lymph nodes were included, and most patients underwent ALND. Therefore, a similar question as concerning the above mentioned monarchE trial remains - are these trial results applicable to patients not undergoing ALND, in which the exact number of nodes is unknown? In the first published TAXIS substudy, TAS removed a median of 5 lymph nodes, 2 of which were positive, whilst ALND removed an additional 14 lymph nodes, 2 of which were positive, thereby totaling 4 positive nodes in the ALND group. Therefore, the TAS only group fell below and the ALND group above the magic line of 3 positive nodes [52].

Moreover, response-driven therapy in patients with residual disease has been shown to enhance oncologic outcomes, both in HER2 positive and triple-negative BC [62,63]. With 70% of patients in the TAXIS trial showing additional nodal disease when undergoing ALND, TAS significantly understaged node-positive patients compared to ALND. However, TAS determined nodal pCR with a FNR of only 2.6% [52].

A planned substudy of the TAXIS trial investigates the influence of known nodal burden after ALND compared to TAS on systemic treatment decisions.

## 9 Morbidity and quality of life

As evidence on oncological safety of de-escalated axillary surgery is continuously provided, also the aspect of morbidity and quality of life is highly relevant, especially in light of long-term breast cancer survivorship. Axillary lymph node dissection is associated with a highly relevant rate of morbidity and decreased QoL.

Morbidity after axillary surgery typically comprises lymphedema and arm swelling, arm abduction deficits and chronic pain and/or sensory loss. The OTOASOR trial provided information of significantly increased rates of compound morbidity (lymphedema, arm swelling, arm pain, paresthesia, and decreased shoulder mobility) in the ALND group (15.3%), compared to the SLNB group (4.7%) after one year. The subgroup of patients receiving ALND followed by RNI showed even higher rates of compound morbidity of 31.5% [11]. In the randomized controlled ALMANAC trial, assigning node-negative patients to either SLNB, with either delayed ALND or axillary radiotherapy if SLN positive, or upfront ALND, quality of life and morbidity were investigated as primary outcomes. Lymphedema occurred significantly more often in patients receiving ALND (moderate or severe: 13% in ALND vs. 5% in SLNB group after 12-months), and sensory deficits were more common after ALND (62% after ALND vs. 16% after SLNB one month postoperatively; and 31% vs. 11% after 12 months). Shoulder motion showed significant differences in flexion and abduction after 1 month, which however was not evident subsequently [64]. Veronesi reported arm swelling ≥1 cm in 37% of patients receiving ALND, and 1% of patients with SLNB after 24 months. Decreased arm mobility <80% was only reported in patients with ALND, and was present in 21%, whilst sporadic (34% vs 7%) and continuous (5% vs. 1%) axillary pain was also more frequent in patients with ALND after 24 months [6]. More recently, lymphedema rates after NST and ALND were investigated in data from the ACOSOG Z1071 trial. Here, in a subset of patients who underwent ALND a cumulative 3-year incidence of 37.8% for self-reported lymphedema symptoms, 58.4% for a 10% arm-volume increase, and 36.9% for a 20% volume increase was shown. Neoadjuvant systemic therapy over 143 days was a significant risk factor for severe lymphedema in multivariate analysis, stressing the need for robust evidence regarding oncological safety of de-escalated axillary surgery [65].

Patients enrolled in the ALMANAC trial showed a reduced QoL and arm function scores at 12-months after ALND [64]. In the NSABP-032 study, patient-reported outcomes showed significantly higher rates of surgery related symptoms, restricted work and social activities, as well as impaired quality of life after ALND in longitudinal analyses, which leveled out after 1 year [66].

## 10 Conclusion

In a series of trials investigating de-escalation of the surgical management of the axilla, the TAXIS trial so far stands out in including cN + patients both after NST as well as in the upfront surgery setting, thereby investigating axillary surgery de-escalation at the far-end of the risk spectrum of node-positive BC patients. The radical Halstedian approach has gradually evolved to an enhanced, oncologically safe, and less-invasive surgery, with TAXIS investigating a novel concept for clinically node positive breast cancer.

## Funding

This work was supported by Agendia, Claudia von Schilling Foundation for Breast Cancer Research, Fond’Action contre le cancer, Kämpf-Bötschi Stiftung, Krebsliga beider Basel, 10.13039/501100016009Rising Tide Foundation for Clinical Cancer Research (RTFCCR), Krebsliga Zentralschweiz, Krebsliga Thurgau, Krebsliga Wallis, Giuliana und Giorgio Stefanini Stiftung, Miaso Stiftung/10.13039/501100009078Hand in Hand Anstalt, Stiftung zur Krebsbekämpfung, 10.13039/100008237Department of Surgery 10.13039/100016015University Hospital Basel, Krebsliga Aargau, Moritz-Straus-Stiftung, Ehmann Foundation Savognin, 10.13039/100009736Freiwillige Akademische Gesellschaft, 10.13039/501100013362Swiss Cancer Research foundation & Swiss Cancer League, Association Marianne Payot, J&K Wonderland Foundation, 10.13039/100009731SANA Fondation, Domarena Stiftung, Fondation pour la Recherche et le Traitement Médical.

No funding sources were involved in the preparation and submission of this manuscript.

## Ethical approval

No ethical approval was required for this work. The TAXIS trial was approved by the local ethics committees and is performed in accordance with the requirements of the national regulatory authorities.

## Declaration of competing interest

The authors declare the following financial interests/personal relationships which may be considered as potential competing interests: W.P. Weber received research support from Agendia paid to the 10.13039/100016015University Hospital Basel for the TAXIS study (OPBC-03, 10.13039/501100011928SAKK 23/16, IBCSG 57–18, ABCSG-53, GBG 101). All other authors declare no competing interests relevant to this manuscript.
